# Endophytic *Beauveria* spp. Enhance Tomato Growth and Resistance to *Botrytis cinerea* via Transcriptomic Regulation

**DOI:** 10.3390/jof11110799

**Published:** 2025-11-10

**Authors:** Yuming Chang, Xiao Lin, Jing Sui, Qiyun Li, Yu Zhao, Li Sui, Zhengkun Zhang

**Affiliations:** 1 Jilin Key Laboratory of Agricultural Microbiology, Institute of Plant Protection, Jilin Academy of Agricultural Sciences, Ministry of Agriculture and Rural Affairs, Gongzhuling 136100, China; ymchang1996@163.com (Y.C.); linxiao66lin@163.com (X.L.); zhaoyu19861104@163.com (Y.Z.); 2 Key Laboratory of Integrated Pest Management on Crops in Northeast China, Institute of Plant Protection, Jilin Academy of Agricultural Sciences, Ministry of Agriculture and Rural Affairs, Gongzhuling 136100, China; 3 College of Horticulture, Jilin Agricultural University, Changchun 130118, China; qyli1225@126.com

**Keywords:** *Solanum lycopersicum*, endophytic colonization, gray mold, differentially expressed genes (DEGs), induced disease resistance, biological control

## Abstract

Entomopathogenic fungi of the genus *Beauveria* are recognized for their dual role as insect pathogens and plant endophytes, however the majority of research efforts to date have centered on *B. bassiana*. To address this bias, we evaluated the endophytic traits of five *Beauveria* species (*B. bassiana*, *B. brongniartii*, *B. aranearum*, *B. amorpha*, and *B. velata*) in tomato (*Solanum lycopersicum*). Tomato seedlings were inoculated by root drenching with 1 × 10^8^ conidia/mL suspensions, and colonization, plant growth, and resistance to *Botrytis cinerea* were assessed. All five species colonized tomato tissues, with colonization rates from 33.3% (*B. velata*) to 56.7% (*B. brongniartii*). Growth promotion was species dependent: *B. bassiana*, *B. brongniartii*, and *B. aranearum* significantly increased plant height, while *B. brongniartii* enhanced aboveground biomass. In pathogen assays, all *Beauveria*-treated plants showed reduced gray mold incidence and severity, with *B. brongniartii* conferring complete protection. Transcriptome analysis identified 160 differentially expressed genes commonly regulated, including 17 upregulated genes enriched in defense responses, hormone signaling, and photosynthesis. These findings demonstrate that non-*B. bassiana* species can establish endophytic associations, promote growth, and induce resistance in tomato, expanding the potential of *Beauveria* spp. as biocontrol agents in sustainable agriculture.

## 1. Introduction

Endophytic fungi are increasingly recognized as key contributors to plant health, promoting growth, enhancing stress tolerance, and strengthening disease resistance through complex symbiotic interactions [1,2]. These endophytes are integral components of the plant microbiota, not only residing within plant tissues but also actively engaging with the rhizosphere microbiome [3]. Through these multifaceted interactions, endophytes influence nutrient cycling, modulate the physicochemical properties of the rhizosphere, and contribute to the assembly of a beneficial microbial community [4,5,6], thereby underpinning overall plant fitness and ecosystem functioning. Within this group, members of the genus *Beauveria* (Hypocreales: Cordycipitaceae) have attracted particular attention due to their dual role as entomopathogens and endophytes, thereby linking pest management with plant protection and positioning them as valuable tools for sustainable agriculture [7,8]. Although the genus encompasses at least 25 recognized species [9], research on endophytic traits has largely centered on *B. bassiana* (Bals.-Criv.) Vuill., the best-characterized member of the group [7]. Numerous studies have demonstrated that *B. bassiana* can successfully colonize a wide range of crops, including tomato [6,10], cucumber [11], rice [12], tobacco [13], maize [14], and wheat [15], via foliar, seed, or soil inoculation [3,16,17]. Such colonization not only promotes plant growth by modulating hormone pathways, such as auxin and gibberellin biosynthesis [18,19] but also induces resistance to phytopathogens like *Botrytis cinerea* Pers. (Helotiales: Sclerotiniaceae; teleomorph: *Botryotinia fuckeliana* (de Bary) Whetzel) (gray mold) through mechanisms including protease inhibitor secretion and the upregulation of defense-related genes [7,20,21]. These findings establish *B. bassiana* as a promising biocontrol agent for integrated pest and disease management.

However, this “*B. bassiana*-centrism” has obscured the broader ecological and functional diversity of the *Beauveria* genus. As emphasized in a comprehensive review by Jaber & Ownley [7], the vast majority of studies on *Beauveria* endophytes focus almost exclusively on *B. bassiana*, with only limited reports on other species. This imbalance has left the endophytic potential of most non-*bassiana* species largely unexplored. More importantly, this narrow focus fundamentally limits the biotechnological potential of the entire genus, as it restricts the discovery of superior strains and the understanding of diverse beneficial mechanisms to a single genetic background. To directly address this knowledge gap and systematically evaluate the untapped functional diversity within the genus, we selected a phylogenetically diverse suite of species for which endophytic traits remain unknown or poorly studied [9,22,23,24]. This includes *B. brongniartii* (Sacc.) Petch (with a single artificial introduction report in broad bean [25]), *B. aranearum* (Petch) Arx, *B. amorpha* (Höhn.) Minnis, S.A. Rehner & Humber, and *B. velata* Samson & H.C. Evans. Our study directly addresses two unresolved questions: (1) Do non-*B. bassiana* species exhibit comparable endophytic colonization capacity in agronomically important crops such as tomato? (2) If so, what biological factors underlie interspecific variation in their growth-promoting and disease-suppressive effects? For these lesser-studied species, knowledge of their ability to colonize host tissues, promote growth, or enhance resistance remains limited. This gap not only restricts our understanding of the genus’s ecological functions but also constrains the development of diverse, eco-friendly biocontrol options. Given the genus’s broad genetic diversity and niche specialization commonly observed in fungal–plant symbioses, it is reasonable to hypothesize that non-*B. bassiana* species may display distinct endophytic traits in tomato, a crop where their potential remains largely unexplored, thereby offering untapped opportunities for agricultural biocontrol.

Notably, tomato (*Solanum lycopersicum* L. (Solanales: Solanaceae)), a globally important vegetable crop, suffers severe yield losses from *B. cinerea*, a necrotrophic pathogen responsible for both preharvest [26] and postharvest [27] damage, thereby threatening worldwide production [28]. Chemical fungicides remain the primary means of control, yet their effectiveness is increasingly undermined by fungicide resistance and environmental concerns [29], highlighting the urgent need for sustainable alternatives such as endophyte-mediated biocontrol [30]. This study investigates five *Beauveria* species for their endophytic colonization in tomato via root drenching, along with their subsequent effects on plant growth, resistance to *B. cinerea*, and the underlying molecular mechanisms via transcriptome analysis. It is anticipated that clarifying the functional diversity among these underutilized species will not only provide a broader palette of candidate strains for designing integrated biological control programs but also shed light on the functional evolution of beneficial traits within the genus *Beauveria*.

## 2. Materials and Methods

### 2.1. Fungal Strains and Preparation of Fungal Inocula

Five *Beauveria* species were used in this study: *B.* bassiana (Bba), *B. brongniartii* (Sacc.) Petch (Bbr), *B. aranearum* (Bar), *B. amorpha* (Bam), and *B. velata* (Bve). The Bba strain BbOFDH1-5-GFP (ACCC No. 32726) was constructed and maintained in our laboratory; its parental strain, BbOFDH1-5 (GenBank accession no.: PRJNA178080), was originally isolated in 2008 from a cadaver of *Ostrinia furnacalis* (Guenée) (Lepidoptera: Pyralidae) at the Institute of Plant Protection, Jilin Academy of Agricultural Sciences, Changchun, China. The remaining four strains including Bbr (ACCC No. 30761), Bar (ACCC No. 30838), Bam (ACCC No. 30834), and Bve (ACCC No. 30876), were obtained from the Agricultural Culture Collection of China (ACCC, Beijing, China). *Botrytis cinerea* (teleomorph: *Botryotinia fuckeliana*), the causal agent of tomato gray mold, was kindly provided by Prof. Wei Li, Hunan Agricultural University, Changsha, China.

All *Beauveria* strains were activated on potato dextrose agar (PDA; Hopebio, Qingdao, China) and incubated at 26 °C in the dark until pale-yellow conidia developed. Mycelial plugs were then transferred to fresh PDA plates for propagation. Aerial conidia from each strain were collected by scraping into sterile centrifuge tubes, suspended in 0.1% (*v*/*v*) sterile Tween-80 (Solarbio, Beijing, China), vortexed for 3 min, and filtered through sterile lens paper (Whatman, Maidstone, UK). Spore concentrations were adjusted to 1 × 10^8^ conidia/mL using a hemocytometer (Marienfeld, Lauda-Königshofen, Germany) and suspensions were stored at 4 °C until use [31]. *Botrytis cinerea*, preserved at −80 °C, was activated by spreading 100 µL of glycerol stock onto PDA plates (Hopebio) and incubating at 26 °C. Once conidia formed, actively growing colonies were sequentially subcultured to the second and third generations to ensure consistent viability for experimental use.

### 2.2. Plant Material and Tomato Seedling Cultivation

Tomato (*Solanum lycopersicum* L.) cv. ‘Jingpin Hongshi Wang’ was obtained from Li Bai Seed Industry Co., Ltd. (Gongzhuling, China). This cultivar, characterized by medium–early maturity and indeterminate growth, is moderately susceptible to *B. cinerea* and was therefore suitable for investigating endophytic colonization and induced disease resistance.

Seeds were surface-sterilized in 1% (*v*/*v*) sodium hypochlorite for 10 min [32], followed by three rinses in sterile distilled water (5 min each) to remove residual disinfectant. Sterilized seeds were then thermally treated by soaking in sterile water at 55 °C, gently stirred until cooled to ~30 °C, and left to soak for an additional 5–6 h to ensure uniform hydration. Treated seeds were placed on sterile Petri dishes lined with moistened filter paper and gauze, and incubated in the dark at 26 °C for germination. Radicle emergence occurred within 2–3 days, with daily addition of sterile water to maintain moisture. Germinated seeds were sown in seedling trays containing sterilized peat soil (Pindstrup Mosebrug A/S, Ryomgård, Denmark), covered with a 1 cm layer of the same soil to prevent compaction, and thoroughly watered. After the appearance of the first true leaves, seedlings were transplanted into 10 × 10 cm pots filled with sterilized growing medium (same source as trays). Plants were irrigated regularly to keep the medium moist but not waterlogged and grown to the 15-day stage (average height ~10 cm) for subsequent inoculation experiments.

### 2.3. Endophytic Colonization of Beauveria *spp.* in Tomato

Root drench inoculation with *Beauveria* strains was performed on 15-day-old tomato seedlings (~10 cm tall) using a randomized block design. Six treatment groups were established, each with three biological replicates per treatment and 10 plants per replicate (total 30 plants per treatment, 180 plants total for the entire experiment). Five groups were treated with suspensions of Bba, Bbr, Bar, Bam, or Bve at 1 × 10^8^ conidia/mL in 0.1% (*v*/*v*) Tween-80, while the control group received only sterile 0.1% Tween-80. For each inoculation, 10 mL of suspension was applied to the soil around the root collar with a sterile 10 mL syringe, avoiding contact with foliage. Inoculations were repeated every other day for a total of three applications to ensure colonization.

At 48 h after the final inoculation, a time point selected to assess early colonization events, colonization was assessed using a PDA assay [33]. One leaf per plant (30 leaves per treatment) was harvested and surface-sterilized under a laminar flow hood by sequential immersion for 30 s in 75% ethanol, 1% sodium hypochlorite, 75% ethanol, and sterile water. Leaf margins were trimmed to reduce contamination, and nine tissue segments (5 × 5 mm) per leaf were blotted dry and placed on PDA plates (Hopebio), followed by incubation at 26 °C. After four days, the presence of *Beauveria* mycelial growth surrounding leaf segments was recorded as confirmation of colonization. A plant was considered colonized if any of its segments exhibited fungal growth. Colonization rate was calculated as:

$$\mathrm{Colonization}\;\mathrm{rate}\;(\%)=\frac{\mathrm{Number}\;\mathrm{of}\;\mathrm{colonized}\;\mathrm{plants}}{\mathrm{Total}\;\mathrm{number}\;\mathrm{of}\;\mathrm{plants}\;\mathrm{per}\;\mathrm{treatment}}\;\times \;100\%$$

### 2.4. Evaluation of Plant Growth-Promoting Effects

Chlorophyll content, nitrogen content, and plant height of all potted plants were measured on the seventh day after the final *Beauveria* inoculation to evaluate growth-promoting effects. Chlorophyll and nitrogen contents in leaves were determined using a chlorophyll meter (Model TYS-B; Zhejiang Top Cloud-Agri Technology Co., Ltd., Hangzhou, China). Plant height was recorded as the distance from the soil surface in the pot to the highest point of the tomato plant.

Following these measurements, three plants were randomly selected from each replicate (9 plants total per treatment). Plants were carefully uprooted, and roots were gently rinsed with sterile water to remove adhering soil particles. Each plant was then divided into aboveground (shoots) and underground (roots) parts at the root–shoot junction. Fresh weights of shoots and roots were measured using an electronic balance with a precision of 0.01 g (Sartorius, Göttingen, Germany). For dry weight determination, shoot and root samples were placed in an oven at 80 °C for 48 h until constant weight was achieved, after which dry weights were recorded.

### 2.5. Assessment of Resistance to B. cinerea

#### 2.5.1. The Confrontation Assays In Vitro

For in vitro confrontation assays, 5 mm mycelial plugs of actively growing *B. cinerea* were excised with a sterile cork borer and placed 1.5 cm from the edge of a PDA plate (Hopebio, Qingdao, China). Opposite each plug, a 5 mm plug of an actively growing *Beauveria* culture was positioned. Plates were incubated at 26 °C in the dark, with six replicates prepared for each *Beauveria* species. Control plates containing only *B. cinerea* (without *Beauveria*) were also included. Plates were monitored for the formation of inhibition zones between the endophyte (*Beauveria*) and the pathogen (*B. cinerea*).

#### 2.5.2. The Resistance Assays In Vivo

For in vivo resistance assays, a separate batch of tomato seedlings was cultivated following the protocol in Section 2.2 to ensure uniform growth. Root drench inoculation with *Beauveria* suspensions was performed as described in Section 2.3 (1 × 10^8^ conidia/mL, 10 mL per plant, applied three times every other day).

The experiment included seven treatment groups with three biological replicates per group and 10 plants per replicate (30 total plants per treatment, 210 total plants for the entire experiment: 7 groups × 3 replicates × 10 plants): (i) control, treated with 10 mL of sterile 0.1% (*v*/*v*) Tween-80 (no *Beauveria* or *B. cinerea*); (ii) pathogen-only group (BC), treated with 10 mL sterile 0.1% (*v*/*v*) Tween-80 followed by *B. cinerea* inoculation; (iii) five *Beauveria* + BC groups, pre-inoculated with Bba, Bbr, Bar, Bam, or Bve suspensions, followed by *B. cinerea* challenge.

At 48 h after the final *Beauveria* inoculation, healthy tomato plants at the four-leaf stage and of uniform vigor were selected for *B. cinerea* inoculation. Pathogen inoculation was performed using 5 mm mycelial plugs excised from the margins (2 cm from the edge) of 4-day-old *B. cinerea* cultures grown at 26 °C. Plugs were placed on the abaxial leaf surface, avoiding major veins, and relative humidity was maintained above 70% to promote infection.

Disease incidence was assessed at 8 days post-inoculation (dpi) and calculated as:

$$\mathrm{Gray}\;\mathrm{mold}\;\mathrm{incidence}\;(\%)\;=\frac{\mathrm{Total}\;\mathrm{number}\;\mathrm{of}\;\mathrm{diseased}\;\mathrm{tomato}\;\mathrm{plants}\;\mathrm{in}\;\mathrm{each}\;\mathrm{treatment}}{\mathrm{Total}\;\mathrm{number}\;\mathrm{of}\;\mathrm{surveyed}\;\mathrm{tomato}\;\mathrm{plants}}\;\times \;100\%$$

Disease severity was rated on a five-class scale as described by Mélida et al. [34]: Grade 1, no symptoms; Grade 2, necrotic spots covering <10% of the leaf area; Grade 3, 11–20%; Grade 4, 21–50%; and Grade 5, >50%. The disease index was calculated as:

$$\mathrm{Disease}\;\mathrm{index}\;=\frac{\sum (\mathrm{Disease}\;\mathrm{grade}\;\mathrm{value}\;\times \;\mathrm{Number}\;\mathrm{of}\;\mathrm{leaves}\;\mathrm{at}\;\mathrm{that}\;\mathrm{grade})}{\mathrm{Highest}\;\mathrm{grade}\;\mathrm{value}\;\times \;\mathrm{Total}\;\mathrm{number}\;\mathrm{of}\;\mathrm{surveyed}\;\mathrm{leaves}}\;\times \;100\%$$

### 2.6. Transcriptome Analysis

Tomato plants from the same batch used for colonization assays (Section 2.3) and growth parameter measurements (Section 2.4) were selected for transcriptome analysis. Sampling was performed 24 h after the final *Beauveria* treatment. For each treatment, nine tomato plants were randomly selected, and the third fully expanded leaf from the top was harvested. Leaf samples were pooled such that every three leaves constituted one biological replicate, resulting in three replicates per treatment. All samples were wrapped in aluminum foil, immediately flash-frozen in liquid nitrogen, and stored at −80 °C until RNA extraction. Total RNA was extracted using the RNAprep Pure Plant Kit (DP441; Tiangen, Beijing, China) according to the manufacturer’s protocol and stored at −80 °C until library construction.

RNA sequencing was conducted on 18 samples (6 groups × 3 replicates), representing three biological replicates from each of the five *Beauveria* treatment groups (Bba, Bbr, Bar, Bam, Bve) and the blank control (162 total plants sampled for the entire transcriptome experiment: 6 groups × 3 replicates × 9 plants). Sequencing libraries were prepared using the NEBNext^®^ Ultra™ RNA Library Prep Kit for Illumina^®^ (New England Biolabs, Ipswich, MA, USA) and sequenced on an Illumina NovaSeq 6000 platform (Illumina, San Diego, CA, USA) at Novogene Bioinformatics Technology Co., Ltd. (Beijing, China) with 150 bp paired-end reads. Raw reads were filtered to remove low-quality sequences (Q20 < 20), adapters, and contaminants. Clean reads were aligned to the tomato reference genome (cultivar: Heinz 1706; accession: GCA_000188115.3, version: SL3.0) using HISAT2 v2.0.5 with default parameters [35]. Gene-level counts were obtained with featureCounts v1.5.0-p3 [36], and expression levels were normalized as fragments per kilobase of transcript per million mapped reads (FPKM). Differentially expressed genes (DEGs) were identified by comparing each *Beauveria* treatment to the control using DESeq2 v1.20.0 [37], with thresholds of adjusted *p* ≤ 0.05 and |log_2_ fold change| ≥ 1. This analysis identified both significantly upregulated and downregulated genes for each treatment compared to the control. All subsequent analyses, including the Venn diagrams and functional enrichment, were performed separately for the upregulated and downregulated DEG sets to discern distinct biological themes associated with induction and suppression of gene expression. No pairwise comparisons were performed among the five *Beauveria* treatments. To visualize shared/unique DEGs across the five *Beauveria* treatments, the Venn diagram was built using 5 DEG sets (each from one Beauveria treatment vs. control) to show overlapping/unique DEG counts and separate upregulated/downregulated subplots.

Gene Ontology (GO) enrichment analysis was carried out using the cluster Profiler R package (version 4.6.2) with Benjamini–Hochberg correction [38], focusing on enriched terms shared across all *Beauveria* vs. control comparisons; GO terms with adjusted *p* < 0.05 were considered significant. Kyoto Encyclopedia of Genes and Genomes (KEGG) pathway analysis was also performed to identify conserved pathways associated with *Beauveria* colonization, with significance defined as adjusted *p* < 0.05. The sequencing data have been deposited at SRA (BioProject ID: PRJNA 1333637).

### 2.7. qRT-PCR Analysis

To validate the reliability of the transcriptomic data, six DEGs were randomly selected from the 17 commonly upregulated DEGs listed in Table 1. These genes were chosen because they were consistently differentially expressed across all five *Beauveria* treatments. Total RNA from the same samples used for transcriptome sequencing (Section 2.6) was retrieved from −80 °C storage and thawed on ice together with the necessary reagents. Reverse transcription was performed to synthesize cDNA using the isolated RNA as template [39]. Primers for the six target DEGs and the reference gene Actin 7 (ACT) [40] were designed with NCBI Primer-BLAST (https://blast.ncbi.nlm.nih.gov/Blast.cgi (accessed on 15 March 2024)), and their sequences are provided in Supplementary Table S1.

Quantitative PCR (qPCR) assays were carried out to determine gene expression levels under different treatments, with the reaction mixture and cycling conditions detailed in Supplementary Tables S2 and S3. Each sample was analyzed with three technical replicates, and non-template controls (RNase-free water) were included to exclude contamination. Relative expression levels were calculated using the 2^−ΔΔCt^ method [41], with ACT serving as the internal reference for normalization.

### 2.8. Statistical Analysis and Visualization

All experimental data, including colonization rates, disease-related parameters (incidence, severity, and disease index), and growth-related traits (plant height, chlorophyll content, nitrogen content, aboveground fresh/dry weight, and underground fresh/dry weight), were analyzed using IBM SPSS Statistics v26.0 (IBM Corp., Armonk, NY, USA). One-way analysis of variance (ANOVA) was conducted to assess overall differences among treatment groups, followed by Duncan’s new multiple range test for pairwise comparisons. Statistical significance was set at *p* < 0.05 for all tests.

Data visualizations for colonization, growth, and disease resistance parameters were generated using GraphPad Prism v8.0.2 (GraphPad Software, San Diego, CA, USA). Bioinformatics-related visualizations were produced using the Bioinfo Intelligent Cloud (BIC) platform (https://www.bic.ac.cn/BIC/#/ (accessed on 3 July 2025)).

## 3. Results

A systematic evaluation of five *Beauveria* species (Bba, Bbr, Bar, Bam, Bve) in tomato revealed distinct endophytic functionalities across four key dimensions. All species colonized tomato tissues at varying rates (33.33–56.67%), with Bbr being the most efficient. Growth promotion was species-specific, evidenced by significant increases in plant height induced by Bba, Bbr, and Bar, and a unique enhancement of aboveground biomass by Bbr. Most notably, all *Beauveria* species reduced gray mold incidence and severity, with Bbr conferring complete protection. Underlying these phenotypes, transcriptomics uncovered a conserved molecular response, including 160 core differentially expressed genes—notably 17 upregulated in defense and photosynthesis—and a coordinated downregulation of hormone signaling pathways.

### 3.1. Endophytic Colonization of Five Beauveria Species in Tomato

**Figure 1:**
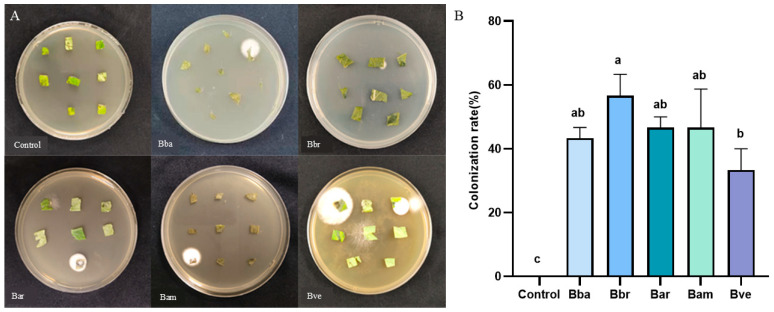
Endophytic colonization of five *Beauveria* species in tomato leaves following root drench inoculation. (**A**) Representative PDA plates showing white mycelial growth around leaf segments colonized by *Beauveria*. (**B**) Colonization rates (%) of tomato by five *Beauveria* species. Values represent mean ± SE (*n* = 30 leaves per treatment). Different letters indicate significant differences among treatments (one-way ANOVA, Duncan’s test, *p* < 0.05).

A clear hierarchy in colonization efficiency was observed among the five *Beauveria* species following root drench inoculation, as confirmed by the development of white mycelia surrounding tomato leaf segments on PDA plates (Figure 1A). Notably, Bbr emerged as the most superior colonizer, achieving a significantly higher colonization rate of 56.67% compared to the least effective species, Bve (33.3%; Figure 1B). The remaining species—Bam (46.67%), Bar (46.67%), and Bba (43.33%)—formed an intermediate group with statistically similar colonization rates. This superior colonization competence of Bbr provides a plausible foundation for its enhanced performance in subsequent growth promotion and disease resistance assays.

### 3.2. Growth-Promoting Effects of Five Beauveria Species on Tomato Plants

The growth-promoting effects of *Beauveria* species on tomato were found to be species-specific. Analysis across the measured variables revealed a clear functional hierarchy: Bbr was identified as the most consistent and comprehensive growth promoter, being the only species that significantly enhanced both plant height and aboveground fresh biomass while maintaining chlorophyll and nitrogen levels comparable to the control. In contrast, Bba and Bar promoted plant height but not biomass, whereas Bam and Bve showed minimal or slightly suppressive effects on multiple growth parameters.

**Figure 2:**
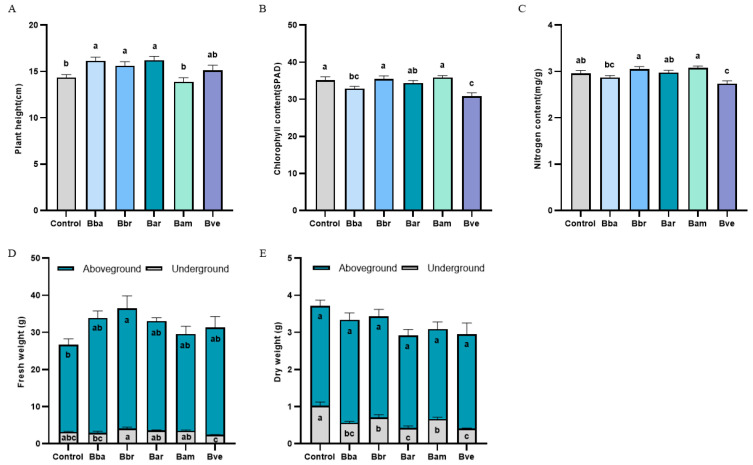
Growth-promoting effects of five *Beauveria* species on tomato plants at 7 dpi. (**A**) Plant height, (**B**) chlorophyll content, (**C**) nitrogen content, (**D**) fresh weights of shoots and roots, and (**E**) dry weights of shoots and roots. Data are presented as mean ± SE (n = 3 biological replicates, 10 plants per replicate). Different letters denote significant differences among treatments (one-way ANOVA, Duncan’s test, *p* < 0.05).

At 7 dpi, plant height was significantly increased by Bba (16.13 cm, +12.56%), Bar (16.22 cm, +13.14%), and Bbr (15.62 cm, +8.98%) compared to the control (14.33 cm, *p* < 0.05; Figure 2A). Bve caused a non-significant increase (15.12 cm), whereas Bam resulted in slightly shorter plants (13.88 cm, *p* > 0.05). Chlorophyll content displayed distinct species-specific patterns (Figure 2B). Bbr, Bar, and Bam maintained levels comparable to the control (34.39–35.84 vs. 35.13 SPAD units). In contrast, Bba and Bve significantly reduced chlorophyll content (*p* < 0.05), with values of 32.89 SPAD units (−6.38%) and 30.83 SPAD units (−12.25%), respectively.

Nitrogen content also varied among treatments (Figure 2C). Bbr, Bar, and Bam slightly increased nitrogen levels (3.05, 2.98, and 3.08 mg/g, +0.68–+ 4.06% vs. control), though not significantly (*p* > 0.05). Bba caused a minor, non-significant decrease (2.87 mg/g, −2.93%), while Bve significantly lowered nitrogen content to 2.93 mg/g (−7.55%, *p* < 0.05).

For aboveground fresh weight (Figure 2D), Bbr promoted the greatest and only statistically significant increase (32.40 g·plant^−1^, +37.90% vs. control 23.49 g·plant^−1^, *p* < 0.05). Although Bba, Bar, Bam, and Bve also showed numerically higher values, these were not significant (*p* > 0.05). For underground fresh weight, none of the treatments resulted in a statistically significant difference compared to the control (*p* > 0.05). Notably, Bbr showed a numerically higher value (4.03 g·plant^−1^, +27.43% vs. control 3.16 g·plant^−1^), suggesting a potential positive trend, whereas Bve exhibited a numerically lower value (2.42 g·plant^−1^, −23.42%), indicating a potential suppressive trend. The values for Bba, Bar, and Bam were intermediate (2.94, 3.60, and 3.52 g·plant^−1^, respectively).

No treatment significantly altered aboveground dry weight (Figure 2E). However, all *Beauveria* treatments significantly reduced underground dry weight relative to the control (1.02 g·plant^−1^, *p* < 0.05). The strongest inhibition occurred with Bar (0.42 g·plant^−1^, −58.82%) and Bve (0.40 g·plant^−1^, −60.78%), followed by Bba (0.57 g·plant^−1^, −44.26%), Bam (0.67 g·plant^−1^, −34.43%), and Bbr (0.71 g·plant^−1^, −30.16%).

### 3.3. Effects of Beauveria Colonization on Resistance to B. cinerea

A consistent trend across both in vitro and in vivo assays was that all *Beauveria* species inhibited *B. cinerea*, but Bbr exhibited exceptional and complete disease protection—a distinction not observed in other species.

**Figure 3:**
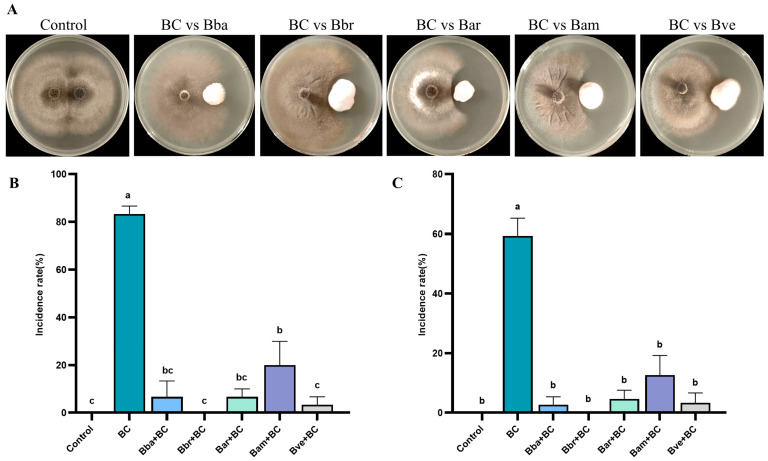
Effects of *Beauveria* spp. colonization on tomato resistance to *B. cinerea*. (**A**) In vitro dual culture confrontation assays showing inhibition of *B. cinerea* growth by *Beauveria*. (**B**) Disease incidence (%) and (**C**) disease index in tomato plants pretreated with different *Beauveria* species and subsequently challenged with *B. cinerea*. Data are expressed as mean ± SE (n = 3 replicates, 10 plants per replicate). Different letters indicate significant differences among treatments (one-way ANOVA, Duncan’s test, *p* < 0.05).

All five *Beauveria* species inhibited the growth of *B. cinerea* in vitro, although their efficacy varied significantly (Figure 3A). Confrontation cultures revealed clear differences in inhibition strength: Bar displayed the strongest activity, forming a pronounced inhibition zone that effectively restricted pathogen expansion, whereas Bba exhibited the weakest suppression, with only a narrow and indistinct inhibition zone.

Consistent with these observations, in vivo assays demonstrated that endophytic colonization by *Beauveria* markedly enhanced tomato resistance to *B. cinerea*, as reflected in both disease incidence and disease index. At 8 dpi, disease incidence was highest in the BC group (83.33%), confirming the strong pathogenicity of *B. cinerea* (Figure 3B). By contrast, all *Beauveria*-pretreated groups showed significantly reduced incidence (*p* < 0.05). Strikingly, the Bbr + BC group completely prevented infection, matching the blank control at 0%. Bve + BC plants showed 3.33% incidence, while Bba + BC and Bar + BC each reached 6.67%. Although Bam + BC recorded the highest incidence among *Beauveria* treatments (20.00%), this still represented a ~76% reduction relative to the BC group.

Disease index results reinforced these patterns (Figure 3C). The BC group reached 59.33%, indicating severe infection, whereas all *Beauveria*-treated groups had significantly lower values (*p* < 0.05). Bbr + BC again provided complete protection (index = 0), while Bba + BC, Bar + BC, and Bve + BC ranged between 2.67% and 4.67%, corresponding to 92.13–95.51% reductions versus BC. Bam + BC, although less effective, still showed a markedly reduced disease index of 12.67% (78.65% reduction).

### 3.4. Transcriptome Analysis of Tomato Responses to Beauveria Colonization

Quality control yielded a total of 114.99 Gb of clean sequencing data, with each sample generating at least 5.8 Gb of high-quality reads (Supplementary Table S4). Sequencing accuracy was high across all 18 samples, with Q20 values (proportion of bases with Phred score ≥ 20) ≥ 97.31% and Q30 values ≥ 92.49%. The average sequencing error rate did not exceed 0.03%, confirming the reliability of the dataset. Mapping statistics further supported data robustness: total reads per sample ranged from 38,639,246 to 49,250,270, of which 33,912,530 to 45,214,543 mapped to the tomato reference genome. Mapping efficiency ranged from 85.48% to 96.29%, with most samples exceeding 90%. Notably, uniquely mapped reads accounted for 85.48–94.50% of total reads, ensuring accurate gene quantification. These metrics established a strong foundation for downstream transcriptomic analyses, including DEG identification and functional enrichment.

**Figure 4:**
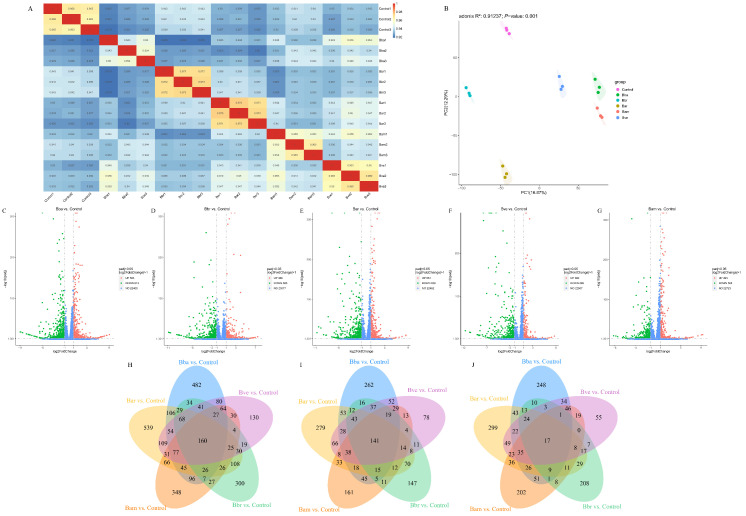
Transcriptome profiling of tomato responses to *Beauveria* colonization. (**A**) Pearson correlation heatmap of gene expression among biological replicates in treatment and control groups. (**B**) Principal component analysis (PCA) showing clustering of transcriptomic profiles. (**C**–**G**) Volcano plots of DEGs in each treatment compared with the control (**C**) Bba; (**D**) Bbr; (**E**) Bar; (**F**) Bam; (**G**) Bve. The *x*-axis represents log_2_ fold change (log_2_FC), with values farther from zero indicating greater expression differences; the *y*-axis represents significance level (−log_10_ padj), with higher values indicating greater significance. (**H**) Venn diagram of DEGs across the five treatments. (**I**) DEGs commonly downregulated in all five treatments. (**J**) DEGs commonly upregulated in all five treatments.

Biological reproducibility was evaluated using Pearson correlation analysis. All treatment and control groups exhibited excellent consistency, with R^2^ values ranging from 0.918 to 0.973 (Figure 4A). Dimensionality reduction analysis further confirmed tight clustering of replicates within each treatment, while groups were clearly separated, reflecting species-specific transcriptional profiles (Figure 4B). Notably, Bba and Bam clustered more closely, suggesting similarity in their regulatory effects. Collectively, these results validated the reproducibility of the experimental design and the biological relevance of between-group differences.

DEGs were identified by comparing each treatment with the control under the criteria padj < 0.05 and |log_2_ fold change| ≥ 1. Volcano plots illustrated the DEG distribution (Figure 4C–G), with the following counts: 1396 (Bba vs. Control), 931 (Bbr vs. Control), 1499 (Bar vs. Control), 1059 (Bam vs. Control), and 949 (Bve vs. Control). In all cases, downregulated genes outnumbered upregulated ones. Venn diagram analysis revealed 160 DEGs consistently dysregulated across treatments, including 17 commonly upregulated and 141 commonly downregulated genes (Figure 4H–J). This conserved transcriptional response, shared by all five endophytic species, provided a plausible molecular basis for the universally observed enhancement in disease resistance. Functional annotation was subsequently focused on the 17 commonly upregulated DEGs because their induction offered the most direct and interpretable mechanistic insight into the activated defense responses, whereas downregulation reflected broader physiological adjustments that were more challenging to link unambiguously to the specific resistance phenotype.

GO and KEGG annotations of the 17 shared upregulated DEGs are summarized in Table 1. These genes primarily clustered into two categories: defense responses and hormone signaling/growth regulation. For defense, Solyc09g084465.1 and Solyc09g089505.1 were enriched in biological processes such as “response to wounding” and “response to stress,” and molecular functions including “endopeptidase inhibitor activity” and “peptidase regulator activity.” Solyc02g065000.1 was annotated with “calcium ion binding” and mapped to the “Plant–pathogen interaction” pathway. The concerted upregulation of these defense-related genes is directly aligned with the significant reduction in gray mold incidence and severity across all *Beauveria* treatments, and may contribute to the complete protection conferred by Bbr. Growth-related DEGs included Solyc11g032100.2 and Solyc03g032190.3, both annotated to the “nucleus,” with the former also linked to “DNA-binding transcription factor activity.” Solyc07g064240.3 was annotated with “electron transfer activity.” The induction of such growth-associated and photosynthesis-related genes correlates with the plant growth promotion, particularly the increased plant height and biomass observed in Bbr-, Bba-, and Bar-treated plants. In addition, Solyc03g083370.3 was enriched in the “lipid metabolic process” category. Six genes lacked clear GO/KEGG annotations, but two (Solyc07g065110.1 and Solyc08g079190.1) were predicted to encode lipid-transfer proteins (LTPs), potentially contributing to host defense.

**Figure 5:**
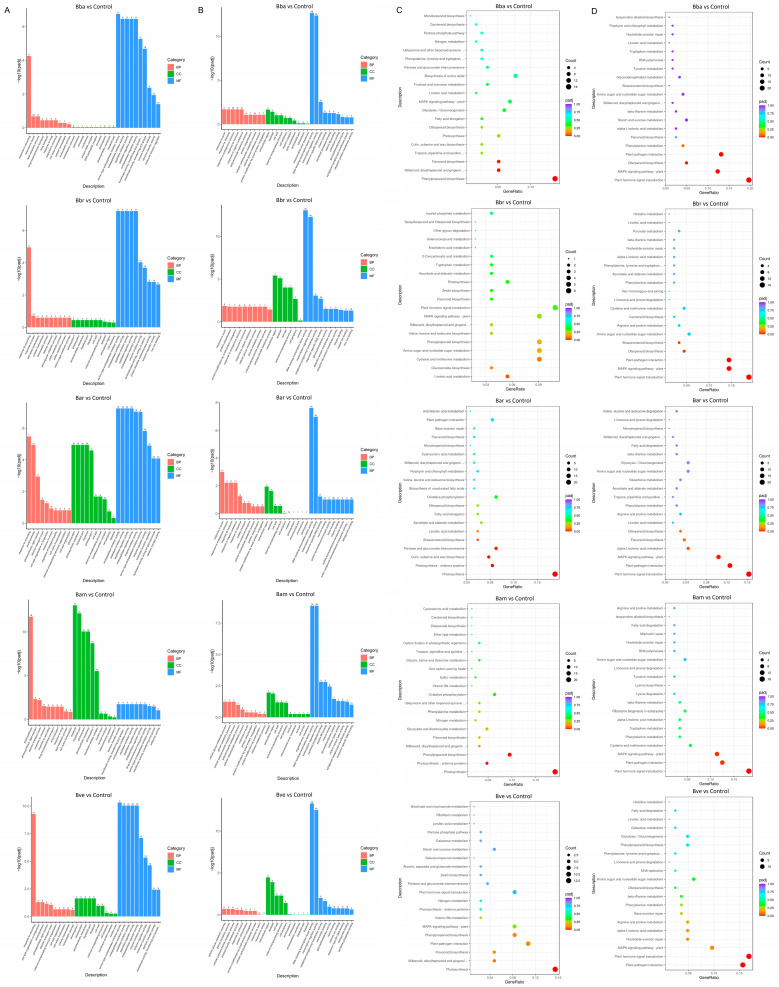
Functional enrichment analysis of differentially expressed genes (DEGs). (**A**) GO enrichment of upregulated DEGs. (**B**) GO enrichment of downregulated DEGs. (**C**) KEGG pathway enrichment of upregulated DEGs. (**D**) KEGG pathway enrichment of downregulated DEGs. For (**A**) and (**B**): the *x*-axis represents GO terms, and the *y*-axis shows enrichment significance (−log_10_ padj). The numbers on top of each bar indicate the number of DEGs annotated to the corresponding GO term. BP, biological process; CC, cellular component; MF, molecular function. For (**C**,**D**): the *x*-axis indicates enrichment ratio (number of DEGs in a pathway vs. total genes annotated to that pathway), and the *y*-axis lists significantly enriched KEGG pathways. Bubble size corresponds to the number of DEGs annotated to each pathway, while bubble color reflects enrichment significance (darker shades = lower adjusted *p*-values).

GO functional analysis of DEGs (adjusted *p* < 0.05) between each *Beauveria* treatment and the control revealed distinct trends for up- and downregulated genes. DEGs were categorized into three main groups: Biological Process (BP), Cellular Component (CC), and Molecular Function (MF). Among upregulated DEGs (Figure 5A), BP terms were enriched in processes such as photosynthesis and response to wounding, consistent with the annotations of Solyc09g084465.1 and Solyc09g089505.1 (Table 1). For CC, the most significantly enriched terms included photosystem, photosystem I, thylakoid, thylakoid part, and photosynthetic membrane. MF enrichment was dominated by peptidase inhibitor activity, peptidase regulator activity, endopeptidase inhibitor activity, endopeptidase regulator activity, and enzyme inhibitor activity. In contrast, downregulated DEGs (Figure 5B) were enriched in BP terms such as response to auxin, cellular glucan metabolic process, protein modification by small protein conjugation, and glucan metabolic process. CC terms included extracellular region, apoplast, cell wall, and external encapsulating structure, while MF terms were mainly DNA-binding transcription factor activity, transcription regulator activity, sequence-specific DNA binding, and vitamin binding.

KEGG pathway analysis further revealed both conserved and species-specific patterns. For upregulated DEGs (Figure 5C), shared significantly enriched pathways included “Photosynthesis” (Bar, Bam, Bve), “Photosynthesis—antenna proteins” (Bar, Bam), and “Phenylpropanoid biosynthesis” (Bba, Bam). Species-specific pathways included “Stilbenoid, diarylheptanoid and gingerol biosynthesis” and “Flavonoid biosynthesis” (Bba), “Linoleic acid metabolism” (Bbr), as well as “Cutin, suberine and wax biosynthesis,” “Pentose and glucuronate interconversions,” and “Brassinosteroid biosynthesis” (Bar). For downregulated DEGs (Figure 5D), three pathways were consistently enriched across all five treatments: “Plant hormone signal transduction,” “MAPK signaling pathway—plant,” and “Plant–pathogen interaction.” The consistent downregulation of these central signaling pathways across all effective *Beauveria* species suggests a conserved host strategy to attenuate hormone signaling, which may mitigate growth–defense trade-offs and contribute to the growth promotion observed in the phenotype, despite the pathogen challenge. Species-specific downregulation was also observed, including “Brassinosteroid biosynthesis” (Bbr), “α-Linolenic acid metabolism” (Bar), and “Diterpenoid biosynthesis” (Bba and Bbr).

### 3.5. qRT-PCR Validation

**Figure 6:**
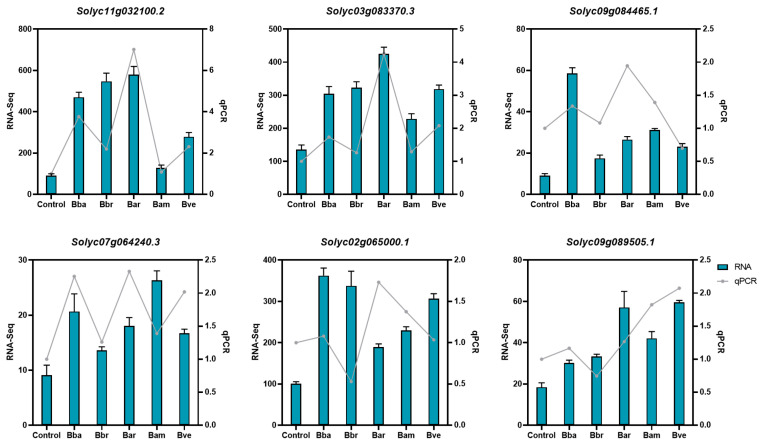
qRT-PCR validation of six DEGs commonly regulated across treatments. Relative expression trends of selected DEGs compared between RNA-Seq and qRT-PCR. Bars represent mean ± SE of three biological replicates, each with three technical replicates.

The expression patterns of six DEGs were highly consistent between RNA-Seq and qRT-PCR analyses, supporting the reliability of the transcriptomic data. As shown in Figure 6, Solyc11g032100.2, Solyc03g083370.3, and Solyc07g064240.3 were upregulated across all treatment groups. Solyc02g065000.1 and Solyc09g089505.1 were upregulated in all treatments except Bbr, while Solyc09g084465.1 was upregulated in all groups but downregulated in Bve.

## 4. Discussion

This study demonstrates that five *Beauveria* species (Bba, Bbr, Bar, Bam, Bve) are capable of colonizing tomato through root drenching, with Bbr exhibiting the highest colonization rate. Among the tested species, Bba, Bar, and Bbr significantly enhanced plant height, while Bbr most effectively increased aboveground biomass. All five species reduced both the incidence and severity of *B. cinerea* infection, with Bbr providing complete protection. Transcriptomic analysis, further validated by qRT-PCR, indicated that enhanced resistance was linked to the upregulation of defense-related genes and photosynthesis-associated pathways, whereas growth promotion appeared to involve modulation of hormone signaling. These findings highlight *Beauveria* species, particularly Bbr, as promising biocontrol agents with potential applications in sustainable tomato production.

This study provides systematic evidence that non-*B. bassiana* species (Bbr, Bar, Bam, Bve) are also capable of establishing endophytic colonization in tomato, thereby expanding our understanding of the ecological versatility of the genus *Beauveria* in plant–fungal interactions. However, a clear and biologically relevant hierarchy in colonization efficiency was observed, ranging from 33.33% (Bve) to 56.67% (Bbr), as it likely reflects intrinsic species-specific traits such as hyphal penetration capacity [17], compatibility with tomato root exudates [42], and competitive ability within the rhizosphere microbiome [43,44], which were selectively assessed by the root drenching method. Critically, this colonization hierarchy was directly correlated with the efficacy in promoting plant growth and, most notably, in inducing disease resistance: species with intermediate colonization rates (Bam; Bar; Bba; 43.33–46.67%) showed moderate beneficial effects, while the superior performance of Bbr—which conferred complete protection against *B. cinerea*—was underpinned by its highest colonization rate. This strong association demonstrates that successful and stable endophytic establishment is a fundamental prerequisite for the full expression of the plant-beneficial functions of these fungi, aligning with the perspective that high and stable colonization is critical for endophytes to exert protective effects [7].

Most *Beauveria* species exerted growth-promoting effects on tomato, with Bba, Bar, and Bbr significantly increasing plant height, and Bbr most effectively enhancing aboveground biomass, findings consistent with previous reports of *B. bassiana*-mediated tomato growth promotion [45]. The transcriptomic data provide a molecular basis for these growth phenotypes. The upregulation of photosynthesis-related pathways and growth-associated genes, such as the nucleus-localized transcription factor Solyc11g032100.2 and Solyc03g032190.3, aligns with the observed increases in plant height and biomass, suggesting enhanced energy capture and cell division [46]. Furthermore, the conserved downregulation of the “Plant hormone signal transduction” pathway across all species may represent a strategic host adjustment to reallocate resources, fine-tuning the balance between growth and subsequent defense investments [47]. Earlier studies have shown that Bba enhances plant growth primarily through improved nutrient acquisition, such as secreting organic acids to solubilize inorganic phosphorus and producing siderophores to chelate Fe^3+^ into absorbable Fe^2+^, as well as through physiological regulation via secretion of phytohormones, including cytokinins and gibberellins, to modulate cell division, elongation, and differentiation [48]. These mechanisms likely contribute to the growth promotion observed here; however, further metabolomic analyses are required to determine whether non-*B. bassiana* species employ similar strategies. An unexpected reduction in underground dry weight across all treatments was consistent with a report on *B. bassiana* [45]. They reported that Bba promotes tomato root growth and water absorption, processes that draw nutrients as plants prioritize allocating resources toward root stress-resilient functions rather than dry matter accumulation. For the non-*B. bassiana* species examined here, a similar reduction in underground dry weight may indicate an analogous resource reallocation strategy. Rather than channeling resources into root stress resilience, as described by Guo et al. [45], our results suggest that resources may instead be prioritized for aboveground disease resistance, although this hypothesis requires confirmation through measurements of root physiological indices.

In vitro confrontation assays confirmed that all *Beauveria* species exhibit direct antagonism against *B. cinerea*, with antifungal activity primarily mediated by secondary metabolites (e.g., oosporein), supplemented by hyphal spatial and physical competition, together suppressing *B. cinerea* growth [49]. In vivo, all *Beauveria* species enhanced tomato resistance to gray mold, with Bbr providing complete protection (0% disease incidence). This protective effect, evidenced by significantly lower disease incidence and index, indicates the induction of systemic resistance, consistent with previous findings [50]. Transcriptome analysis supports this dual defense mechanism of “direct antagonism + induced resistance.” The conserved upregulation of a core set of defense genes, including the wound-induced protease inhibitors Solyc09g084465.1 and Solyc09g089505.1, likely directly neutralizes *B. cinerea* virulence factors. Rathi et al. [51] reported that *B. cinerea* secretes effectors such as BcSnod1 and BcSnod2 to trigger necrosis in tomato leaves; the protease inhibitors upregulated here likely target these effectors, thereby mitigating cellular damage. This interpretation is consistent with previous findings that tomato activates a suite of defense genes, including protease inhibitors, upon pathogen challenge, which act both locally at infection sites and systemically in uninfected tissues to strengthen plant defense [52]. The induction of other defense-related genes further highlights a broad activation of immune signaling. For instance, Solyc02g065000.1, annotated with calcium ion binding activity and mapped to the “Plant–pathogen interaction” pathway, may function as a second messenger to transmit pathogen-derived signals and activate tomato defenses [53]. Solyc03g083370.3, enriched in lipid metabolic processes, has an *Arabidopsis* homolog implicated in ethylene signaling and systemic resistance [54,55]. Given its links to lipid metabolism and signal transduction [56,57], this gene may contribute to both growth promotion and enhanced resistance in tomato. The conserved downregulation of central signaling pathways suggests a shared host strategy to optimize immunity. The attenuation of “Plant hormone signal transduction” and “MAPK signaling” may prevent the potential negative effects of hormone overactivation on defense, a phenomenon observed in other plant–pathogen systems where hyperactivated jasmonic acid or abscisic acid signaling can suppress immunity [58,59,60]. The distinctively complete protection conferred by Bbr may be further attributed to its unique upregulation of the “Linoleic acid metabolism” pathway, warranting targeted metabolite profiling to elucidate underlying mechanisms.

Finally, the reliability of the transcriptomic data is supported by the cross-species validation of a core transcriptional signature. The use of a |log_2_FC| ≥ 1 threshold, a widely adopted standard [60,61], reproducibly identified a common set of DEGs across five phylogenetically distinct species, strongly indicating the capture of a robust, biologically relevant response rather than stochastic noise. This provides a high-confidence foundation for the mechanistic interpretations presented. Notably, six of the 17 commonly upregulated DEGs remain unannotated, hinting at novel regulatory mechanisms in *Beauveria*–tomato interactions that merit future functional validation.

## 5. Conclusions

In conclusion, this study highlights the largely untapped potential of non-*B. bassiana Beauveria* species as effective biocontrol agents following endophytic colonization. Among them, *B. brongniartii* stands out as a particularly promising candidate due to its high colonization rate, strong growth-promoting effects, and unique ability to confer complete protection against disease. Furthermore, the conserved genes and pathways identified across treatments provide valuable insights into the molecular basis of *Beauveria*–host interactions.

Looking forward, the efficacy of *B. brongniartii* warrants its development into a novel, multi-functional bio-inoculant for sustainable agriculture. Future research should prioritize field validation and focus on elucidating the functional roles of the core conserved genes, particularly the unannotated ones, and the secondary metabolites underlying its superior performance. Furthermore, our findings advocate for a paradigm shift in biocontrol screening towards evaluating phylogenetic diversity within a genus, which can unlock a broader spectrum of ecological functions beyond those of a single model species.

## Figures and Tables

**Table 1:** GO and KEGG annotations of 17 commonly upregulated DEGs induced by *Beauveria* colonization in tomato.

Gene ID	Gene Description	GO Category	GO ID	GO Annotation	KEGG ID	KEGG Pathway Annotation
Solyc01g106310.3	Not annotated	Not annotated	Not annotated	Not annotated	Not annotated	Not annotated
Solyc11g032100.2	Agamous-like MADS-box protein AGL12	Cellular Component (CC)	GO:0005634	nucleus	Not annotated	Not annotated
		Molecular Function (MF)	GO:0003700; GO:0140110; GO:0046983	DNA-binding transcription factor activity; transcription regulator activity; protein dimerization activity	Not annotated	Not annotated
Solyc03g032190.3	G2/mitotic-specific cyclin-1	CC	GO:0005634	nucleus	Not annotated	Not annotated
novel.1187	Not annotated	Not annotated	Not annotated	Not annotated	Not annotated	Not annotated
Solyc07g065110.1	Putative lipid-transfer protein DIR1	Not annotated	Not annotated	Not annotated	Not annotated	Not annotated
Solyc03g083370.3	GDSL esterase/lipase At4g10955	Biological Process (BP)	GO:0006629	lipid metabolic process	Not annotated	Not annotated
Solyc06g007890.3	Not annotated	Not annotated	Not annotated	Not annotated	Not annotated	Not annotated
Solyc08g079190.1	pEARLI1-like lipid transfer protein 2	Not annotated	Not annotated	Not annotated	Not annotated	Not annotated
Solyc09g084465.1	Wound-induced proteinase inhibitor 1	BP	GO:0009611; GO:0006950	response to wounding; response to stress	Not annotated	Not annotated
		MF	GO:0004866; GO:0030414; GO:0061134; GO:0061135; GO:0004867; GO:0004857; GO:0030234; GO:0098772	endopeptidase inhibitor activity; peptidase inhibitor activity; peptidase regulator activity; endopeptidase regulator activity; serine-type endopeptidase inhibitor activity; enzyme inhibitor activity; enzyme regulator activity; molecular function regulator	Not annotated	Not annotated
Solyc01g066910.3	Not annotated	Not annotated	Not annotated	Not annotated	Not annotated	Not annotated
Solyc07g064240.3	Early nodulin-like protein 3	MF	GO:0009055	electron transfer activity	Not annotated	Not annotated
Solyc05g053850.3	Protein FLOWERING LOCUS T	Not annotated	Not annotated	Not annotated	Not annotated	Not annotated
Solyc02g065000.1	Calmodulin-like protein 1	MF	GO:0005509	calcium ion binding	sly04626	Plant–pathogen interaction
Solyc09g089505.1	Proteinase inhibitor I-B	BP	GO:0009611; GO:0006950	response to wounding; response to stress	Not annotated	Not annotated
		MF	GO:0004866; GO:0030414; GO:0061134; GO:0061135; GO:0004867; GO:0004857; GO:0030234; GO:0098772	endopeptidase inhibitor activity; peptidase inhibitor activity; peptidase regulator activity; endopeptidase regulator activity; serine-type endopeptidase inhibitor activity; enzyme inhibitor activity; enzyme regulator activity; molecular function regulator	Not annotated	Not annotated
Solyc01g091040.3	Cysteine-rich repeat secretory protein 15	Not annotated	Not annotated	Not annotated	Not annotated	Not annotated
Solyc03g096050.3	Protein DOWNY MILDEW RESISTANCE 6	Not annotated	Not annotated	Not annotated	Not annotated	Not annotated
Solyc05g051450.2	Not annotated	Not annotated	Not annotated	Not annotated	Not annotated	Not annotated

## Data Availability

The data presented in this study are openly available in the Sequence Read Archive (SRA) of the National Center for Biotechnology Information (NCBI) at https://www.ncbi.nlm.nih.gov/bioproject/1333637 (accessed on 24 September 2025), reference number PRJNA1333637.

## Supplementary Materials

The following supporting information can be downloaded at: https://www.mdpi.com/article/10.3390/jof11110799/s1, Table S1: Primer Information for qRT-PCR; Table S2: qPCR Reaction System (Total volume: 20 μL); Table S3: Thermal Cycling Conditions for qRT-PCR; Table S4: RNA-Seq data quality and genome mapping statistics.
