# Supplementary material for: Endophytic Beauveria spp. Enhance Tomato Growth and Resistance to Botrytis cinerea via Transcriptomic Regulation
Source: J Fungi (Basel). 2025 Nov 10;11(11):799. doi: 10.3390/jof11110799 (PMC12653444; doi:10.3390/jof11110799)
Supplement: Supplementary file 1 [file jof-11-00799-s001.zip › jof-3932767-supplementary.pdf]

Journal of *Fungi*

MDPI

Supplementary Materials

Table S1. Primer Information for qRT-PCR.

Gene ID	Primer sequence (5′to 3′)	Fragment length (bp)
Actin 7 (*ACT*)	F:GGTATCCACGAGACTACCTACA<br>R:TGCTCATACGGTCAGCAATAC	127
Solyc11g032100.2	F:ATCTTGGTGCAAAGGAAAACAA<br>R:CATCGAAAATCCTTTGTGACTGG	147
Solyc03g083370.3	F:AGACTCTCATGGCACGAAAAGA<br>R:CAATAAGTTCTGGCCACATTCGT	111
Solyc09g084465.1	F:TCAAAGCTCTGTAAATTGGGGGT<br>R:GGCACCACAATTCTACACGTG	188
Solyc07g064240.3	F:AGCTTCTGATGTACCTGCCG<br>R:TAGCCAAAGTGCCATGATTAGAG	158
Solyc02g065000.1	F:GTATTCGGCGGCTCCCTATT<br>R:TCGGCCAAAATAACCATGAGC	159
Solyc09g089505.1	F:ATTTCGCTGTAGGGGGTTGG<br>R:AGAAACCGCGGCAGTTAAGA	113

Table S2. qPCR Reaction System (Total volume: 20 μL).

Component	Volume
2×ChamQ Universal SYBR qPCR Master Mix	10 μL
Forward primer (10 μM)	0.4 μL
Reverse primer (10 μM)	0.4 μL
Diluted cDNA template	2 μL
RNase-free water	7.2 μL

Table S3. Thermal Cycling Conditions for qRT-PCR.

Stage	Temperature	Duration
Initial denaturation	95°C	30 sec
Amplification (40 cycles):		
Denaturation	95°C	10 sec
Annealing/Extension	60°C	30 sec
Dissociation stage：		
Step 1	95°C	15 sec
Step 2	60°C	1 min
Step 3	95°C	15 sec

 https://doi.org/10.3390/jof11110799

Table S4. RNA-Seq data quality and genome mapping statistics.

Sample	Raw reads	Clean reads (Gb)	Clean bases	Error rate (%)	Q20 (%)	Q30 (%)	Total mapped reads	Uniquely mapped reads	Multiply mapped reads	Read 1 mapped	Read 2 mapped
Control1	50,056,644	49,250,270	7.39G	0.03	97.53	92.86	45,214,543 (91.81%)	44,421,482 (90.20%)	793,061 (1.61%)	22,261,236 (45.20%)	22,160,246 (45.00%)
Control2	43,354,812	42,232,478	6.33G	0.02	98.24	94.58	39,124,431 (92.64%)	38,443,645 (91.03%)	680,786 (1.61%)	19,198,239 (45.46%)	19,245,406 (45.57%)
Control3	43,762,084	42,234,986	6.34G	0.03	97.82	93.56	38,881,397 (92.06%)	38,224,389 (90.50%)	657,008 (1.56%)	19,131,237 (45.30%)	19,093,152 (45.21%)
Bba1	44,904,244	43,983,034	6.6G	0.03	97.31	92.49	40,887,331 (92.96%)	40,209,131 (91.42%)	678,200 (1.54%)	20,156,243 (45.83%)	20,052,888 (45.59%)
Bba2	40,032,492	39,000,960	5.85G	0.02	98.16	94.44	33,988,594 (87.15%)	33,339,891 (85.48%)	648,703 (1.66%)	16,657,892 (42.71%)	16,681,999 (42.77%)
Bba3	47,130,396	46,122,510	6.92G	0.03	97.49	92.88	41,899,460 (90.84%)	41,181,285 (89.29%)	718,175 (1.56%)	20,639,687 (44.75%)	20,541,598 (44.54%)
Bbr1	45,187,830	44,548,688	6.68G	0.03	97.42	92.63	40,069,350 (89.95%)	39,273,392 (88.16%)	795,958 (1.79%)	19,706,890 (44.24%)	19,566,502 (43.92%)
Bbr2	42,802,726	41,993,278	6.3G	0.02	98.25	94.62	38,389,665 (91.42%)	37,627,596 (89.60%)	762,069 (1.81%)	18,801,475 (44.77%)	18,826,121 (44.83%)
Bbr3	42,925,344	42,126,714	6.32G	0.03	97.60	93.12	38,196,067 (90.67%)	37,450,316 (88.90%)	745,751 (1.77%)	18,777,293 (44.57%)	18,673,023 (44.33%)
Bar1	43,106,484	42,305,562	6.35G	0.03	97.61	93.04	40,627,672 (96.03%)	39,865,073 (94.23%)	762,599 (1.80%)	19,971,094 (47.21%)	19,893,979 (47.02%)
Bar2	48,296,334	46,694,760	7.0G	0.02	98.16	94.4	44,963,158 (96.29%)	44,125,589 (94.50%)	837,569 (1.79%)	22,053,609 (47.23%)	22,071,980 (47.27%)
Bar3	46,004,510	44,304,816	6.65G	0.03	97.79	93.51	42,526,730 (95.99%)	41,761,964 (94.26%)	764,766 (1.73%)	20,897,663 (47.17%)	20,864,301 (47.09%)
Bam1	40,439,612	39,812,966	5.97G	0.03	97.42	92.67	36,612,744 (91.96%)	35,942,519 (90.28%)	670,225 (1.68%)	18,013,881 (45.25%)	17,928,638 (45.03%)
Bam2	42,944,534	42,121,456	6.32G	0.02	98.26	94.53	36,988,218 (87.81%)	36,209,906 (85.97%)	778,312 (1.85%)	18,095,096 (42.96%)	18,114,810 (43.01%)
Bam3	40,763,426	38,639,246	5.8G	0.03	97.84	93.62	33,912,530 (87.77%)	33,233,816 (86.01%)	678,714 (1.76%)	16,624,616 (43.03%)	16,609,200 (42.99%)
Bve1	41,035,446	40,290,756	6.04G	0.03	97.51	92.88	38,432,621 (95.39%)	37,774,459 (93.75%)	658,162 (1.63%)	18,927,547 (46.98%)	18,846,912 (46.78%)
Bve2	41,970,744	40,945,678	6.14G	0.02	98.1	94.24	39,239,968 (95.83%)	38,524,096 (94.09%)	715,872 (1.75%)	19,259,379 (47.04%)	19,264,717 (47.05%)
Bve3	40,807,644	39,903,304	5.99G	0.03	97.65	93.27	37,940,949 (95.08%)	37,260,329 (93.38%)	680,620 (1.71%)	18,667,416 (46.78%)	18,592,913 (46.59%)

Notes: Q20 and Q30 represent the percentage of bases with Phred quality scores ≥20 and ≥30, respectively. Mapping values are expressed as percentages of clean reads.
